# Supplementary figures and images for: Diversity structure of the microbial communities in the guts of four neotropical termite species
Source: PeerJ. 2021 Apr 7;9:e10959. doi: 10.7717/peerj.10959 (PMC8035897; doi:10.7717/peerj.10959)

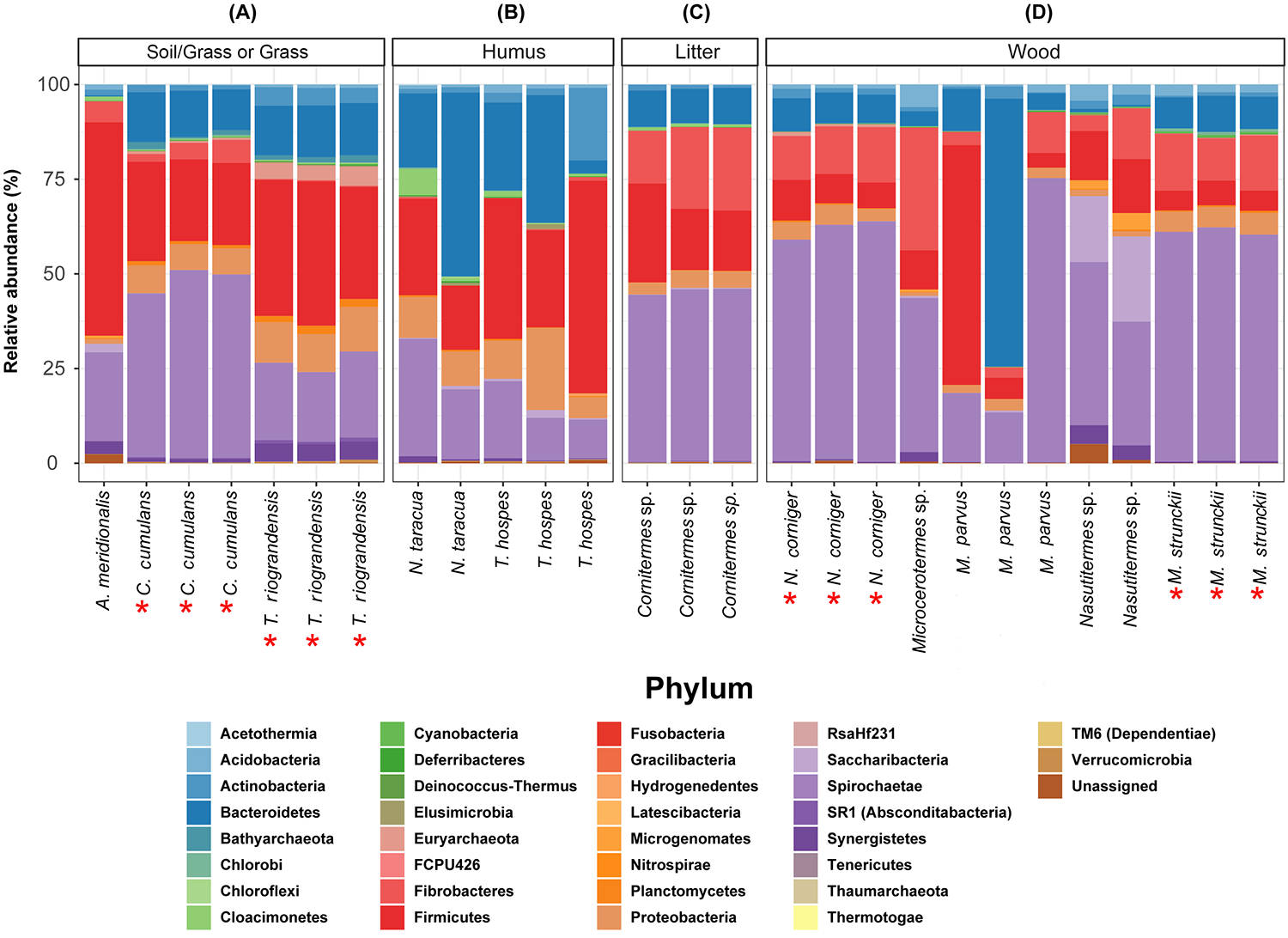

Supplement: Supplemental Information 1 [file peerj-09-10959-s001.png]

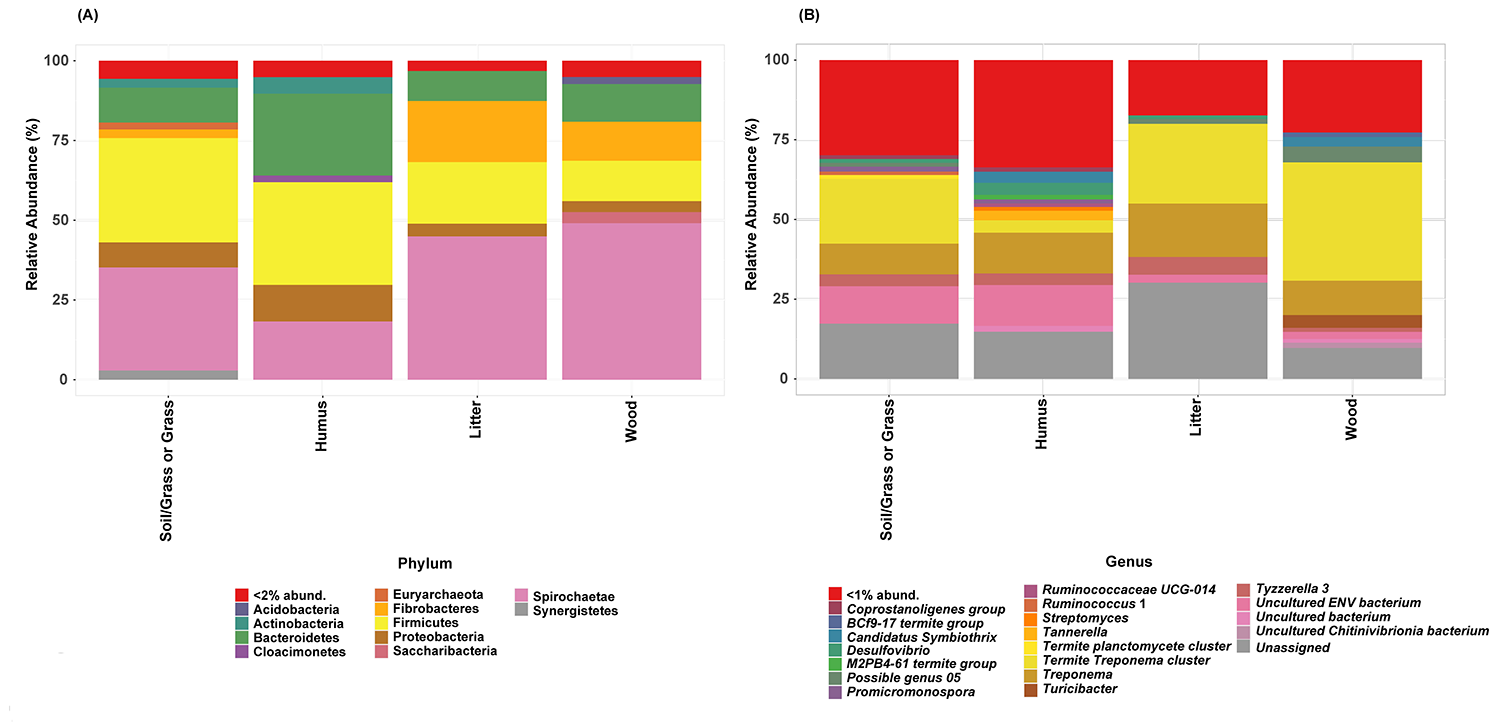

Supplement: Supplemental Information 2 — (A). Phylum level taxonomic classification (B) Genus level taxonomic classification. Taxa less than 2% were combined and named as a “<2% abund.”. [file peerj-09-10959-s002.png]

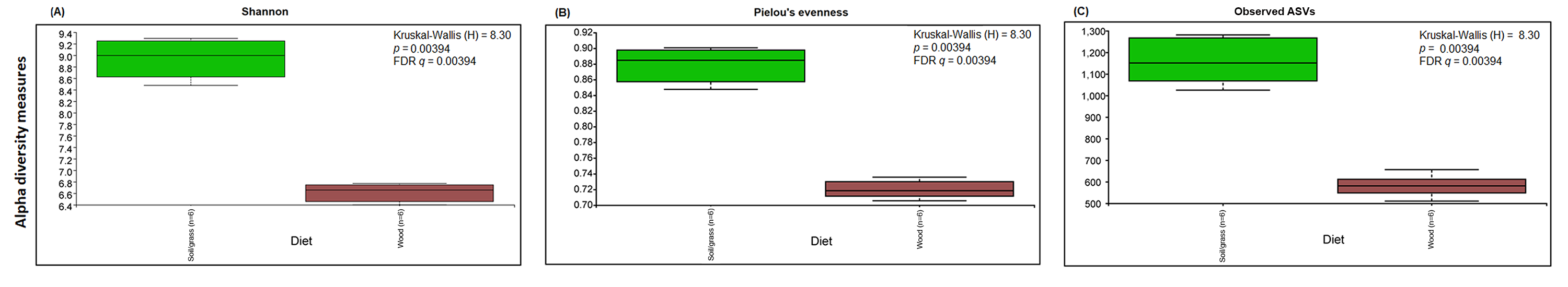

Supplement: Supplemental Information 3 — Comparisons were performed using the Kruskal-Wallis followed by FDR method in Qiime2. [file peerj-09-10959-s003.png]

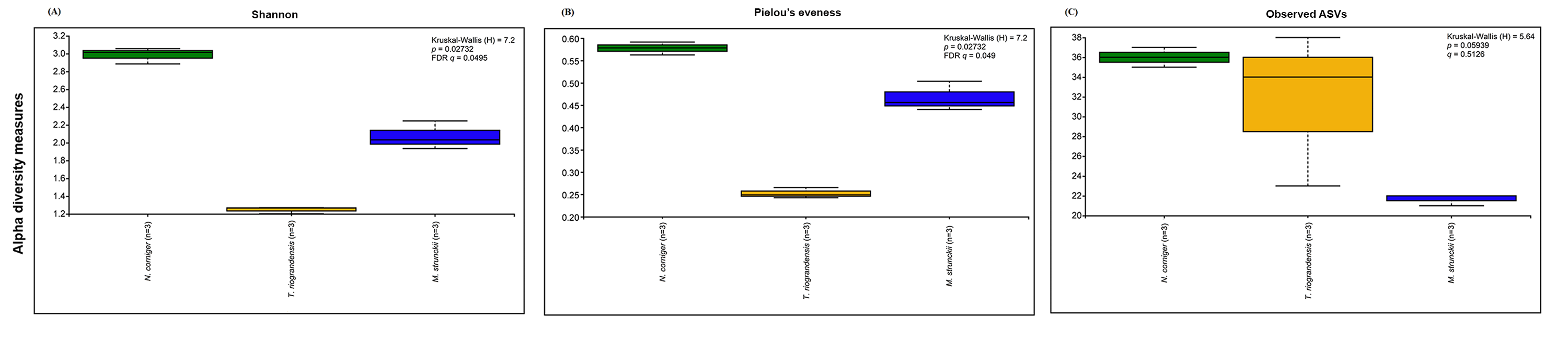

Supplement: Supplemental Information 4 — Comparisons were performed using Kruskal-Wallis followed by FDR method in Qiime2. [file peerj-09-10959-s004.png]

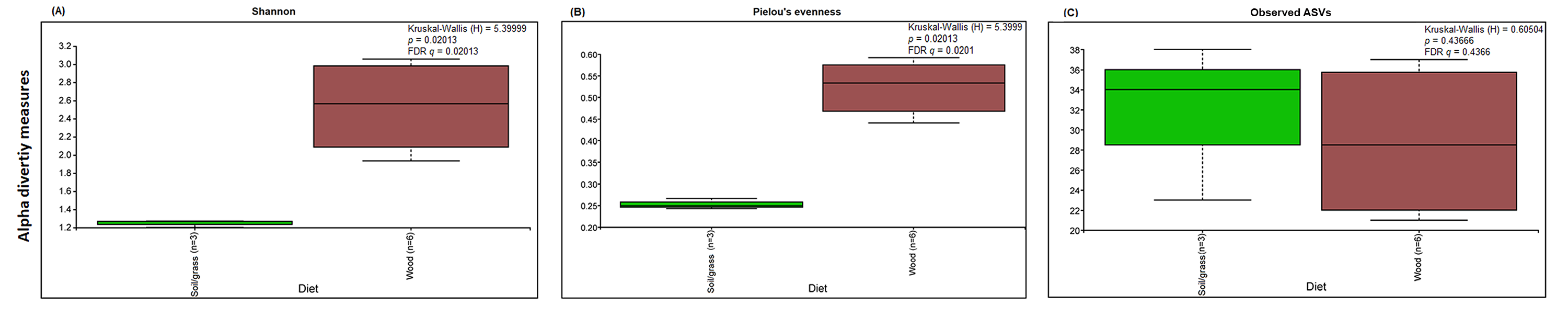

Supplement: Supplemental Information 5 — Comparisons were performed using Kruskal-Wallis followed by FDR method in Qiime2. [file peerj-09-10959-s005.png]

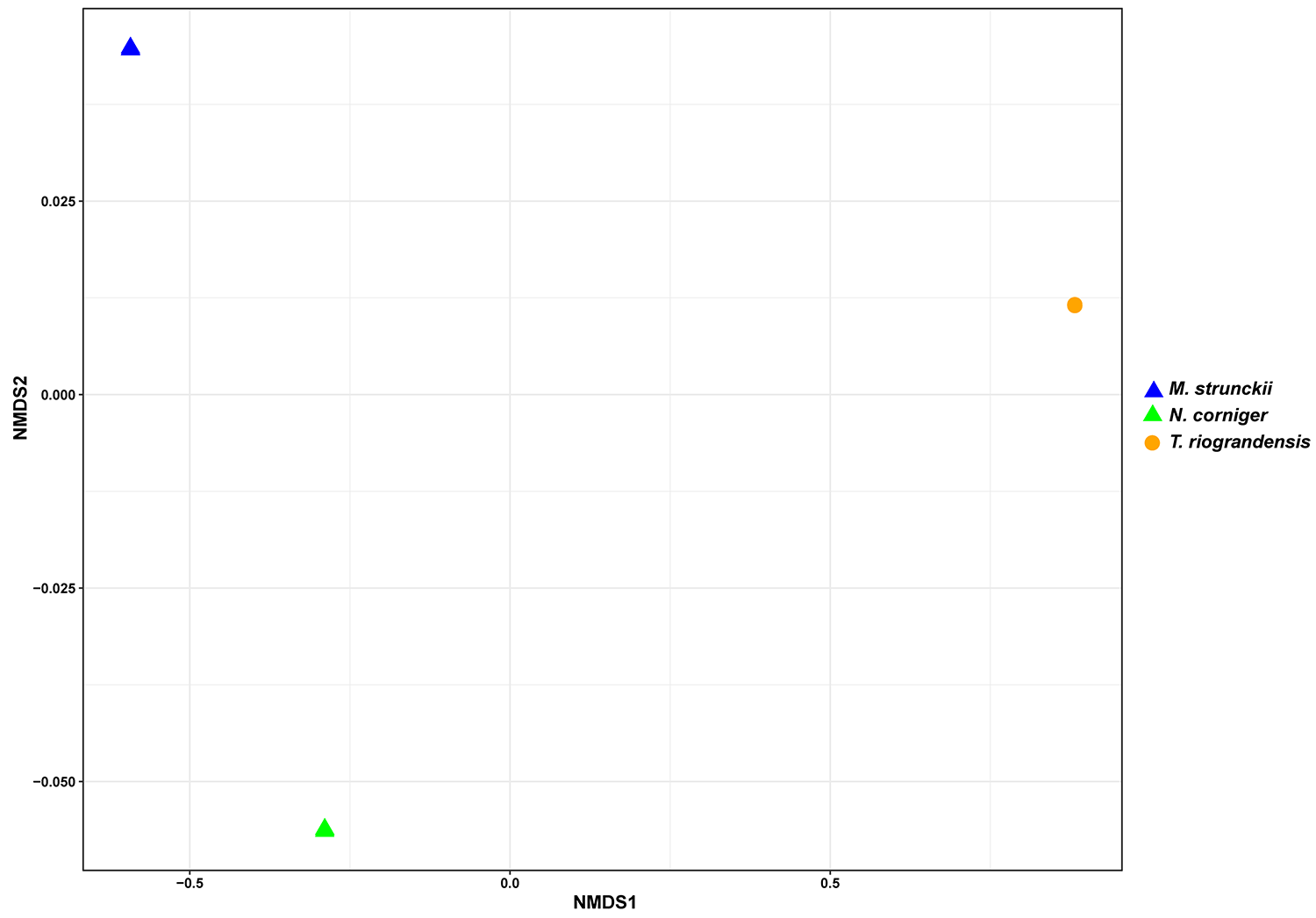

Supplement: Supplemental Information 6 [file peerj-09-10959-s006.png]

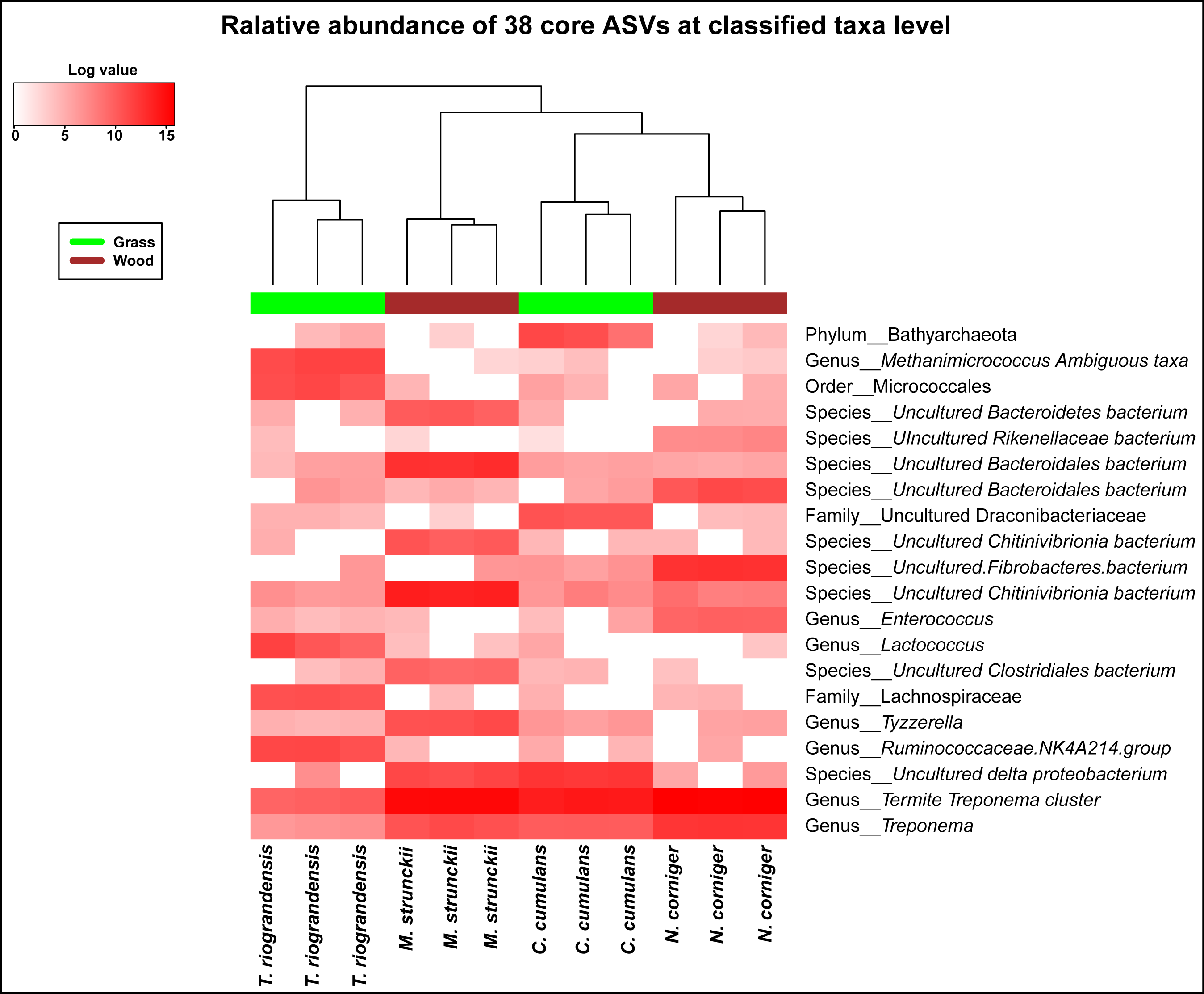

Supplement: Supplemental Information 7 [file peerj-09-10959-s007.png]
