# Supplementary material for: Diversity structure of the microbial communities in the guts of four neotropical termite species
Source: PeerJ. 2021 Apr 7;9:e10959. doi: 10.7717/peerj.10959 (PMC8035897; doi:10.7717/peerj.10959)
Supplement: Supplemental Information 8 [file peerj-09-10959-s008.docx]

**Supplementary Table S1A**

| Termite Sample | 16S reads | | ITS reads | |
| --- | --- | --- | --- | --- |
|  | **Input reads** | **Retained reads** | **Input reads** | **Retained reads** |
| *N. corniger_*1 | 117,868 | 101,995 | 70,498 | 7,418 |
| *N. corniger*_2 | 116,523 | 99,811 | 73,149 | 7,777 |
| *N. corniger_3* | 98,867 | 87,738 | 65,260 | 7,811 |
| *C. cumulans_*1 | 101,453 | 85,051 | 3,435 | 1,496 |
| *C. cumulans_*2 | 97,101 | 83,257 | 2,709 | 1,751 |
| *C. cumulans*_3 | 91,156 | 79,256 | 3,213 | 2,127 |
| *T. riograndensis_*1 | 113,665 | 99,074 | 38,375 | 33,715 |
| *T. riograndensis_*2 | 97,203 | 87,870 | 46,435 | 40,435 |
| *T. riograndensis_*3 | 100,776 | 90,828 | 39,888 | 34,887 |
| *M. strunckii_*1 | 98,197 | 88,304 | 76,068 | 66,472 |
| *M. strunckii_*2 | 97,526 | 87,204 | 72,017 | 62,588 |
| *M. strunckii_*3 | 108,580 | 96,445 | 80,370 | 70,889 |

**Supplementary Table S1B**

| Termite Sample | Input  reads | Retained reads |
| --- | --- | --- |
| *Amitermis meridionalis* | 1,6036 | 14,042 |
| *N. corniger*_1 | 117,868 | 107,234 |
| *N. corniger*_2 | 116,523 | 105,686 |
| *N. corniger*_3 | 98,867 | 89,888 |
| *Cornitermes*_1 | 70,457 | 24,482 |
| *Cornitermes*_2 | 406,809 | 160,064 |
| *Cornitermes*_3 | 406,809 | 160,064 |
| *C. cumulans*_1 | 101,453 | 86,591 |
| *C. cumulans*_2 | 97,101 | 84,081 |
| *C. cumulans*_3 | 91,156 | 79,172 |
| *Microcerotermes* sp._E_1 | 16,924 | 15,957 |
| *Microcerotermes* parvus_2 | 248,700 | 113,417 |
| *Microcerotermes* parvus_3 | 117,671 | 47,489 |
| *Microcerotermes* parvus_4 | 331,534 | 120,712 |
| *Nasutitermes*_sp._1 | 15,020 | 14,037 |
| *Nasutitermes*_sp._2 | 19,103 | 17,420 |
| *Neocapritermes taracua*_1 | 23,198 | 8,210 |
| *Neocapritermes taracua*_2 | 53,083 | 24,830 |
| *Neocapritermes taracua*_3 | 26,116 | 14,022 |
| *T. riograndensis*_1 | 113,665 | 100,418 |
| *T. riograndensis*_2 | 97203 | 87771 |
| *T. riograndensis*_3 | 100776 | 90620 |
| *M. strunkii*_1 | 98197 | 92305 |
| *M. strunkii*_2 | 97526 | 91546 |
| *M. strunkii*_3 | 108580 | 101713 |
| *Thermes hospes*_1 | 43870 | 21297 |
| *Thermes hospes*_2 | 34491 | 14398 |
| *Thermes hospes*_3 | 128014 | 47039 |
