# Supplementary material for: Diversity structure of the microbial communities in the guts of four neotropical termite species
Source: PeerJ. 2021 Apr 7;9:e10959. doi: 10.7717/peerj.10959 (PMC8035897; doi:10.7717/peerj.10959)
Supplement: Supplemental Information 9 [file peerj-09-10959-s009.docx]

| Domain | Phylum | Class/Family | Genus or lowest classification available | Number of ASVs in taxon | Average abundance (%) | *C. cumulans*  reads (%) | *M. strunckii* reads (%) | *N. corniger* reads (%) | *T. riograndensis* reads (%) |
| --- | --- | --- | --- | --- | --- | --- | --- | --- | --- |
| Bacteria | Spirochaetae | Spirochaetaceae | Termite Treponema cluster | 15 | 17.70 | 11.35 | 24.90 | 33.79 | 0.58 |
|  | Spirochaetae | Spirochaetaceae | Treponema | 3 | 1.33 | 0.68 | 1.21 | 3.71 | 0.06 |
|  | Firmicutes | Lachnospiraceae | Tyzzerella | 1 | 0.33 | 0.04 | 1.24 | 0.02 | 0.01 |
|  | Firmicutes | Ruminococcaceae | Ruminococcaceae NK4A214 group | 1 | 0.38 | 0.01 | 0.004 | 0.009 | 1.48 |
|  | Firmicutes | Streptococcaceae | Lactococcus | 1 | 0.30 | 0.009 | 0.006 | 0.002 | 1.17 |
|  | Firmicutes | Enterococcaceae | Enterococcus | 1 | 0.14 | 0.01 | 0.004 | 0.51 | 0.01 |
|  | Firmicutes | Clostridiales | uncultured Clostridiales bacterium | 1 | 0.12 | 0.01 | 0.44 | 0.002 | 0.009 |
|  | Firmicutes | Clostridiales | Lachnospiraceae | 1 | 0.28 | 0.006 | 0.004 | 0.01 | 1.11 |
|  | Bacteroidetes | Rikenellaceae | uncultured Bacteroidales bacterium | 2 | 1.49 | 0.06 | 4.57 | 1.26 | 0.06 |
|  | Bacteroidetes | COB P4-1 termite group | uncultured Bacteroidales bacterium | 1 | 0.18 | 0.006 | 0.68 | 0.01 | 0.01 |
|  | Bacteroidetes | Marinilabiaceae | uncultured Rikenellaceae bacterium | 1 | 0.03 | 0.0008 | 0.001 | 0.09 | 0.003 |
|  | Bacteroidetes | Draconibacteriaceae |  | 1 | 0.23 | 0.91 | 0.001 | 0.007 | 0.01 |
|  | Fibrobacteres | Fibrobacteraceae | uncultured Fibrobacteres bacterium | 2 | 1.12 | 0.05 | 0.018 | 4.36 | 0.01 |
|  | Fibrobacteres | Chitinivibrionia Incertae Sedis | uncultured Chitinivibrionia bacterium | 1 | 0.20 | 0.10 | 0.76 | 0.008 | 0.006 |
|  | Fibrobacteres | possible family 02 | uncultured Chitinivibrionia bacterium | 2 | 0.05 | 0.009 | 8.94 | 0.21 | 0.05 |
|  | Proteobacteria | Deltaproteobacteria | uncultured delta proteobacterium | 1 | 1.29 | 3.50 | 1.60 | 0.024 | 0.02 |
|  | Actinobacteria | Micrococcales |  | 1 | 0.35 | 0.01 | 0.005 | 0.01 | 1.35 |
| Archaea | Bathyarchaeota |  |  | 1 | 0.27 | 1.06 | 0.001 | 0.005 | 0.01 |
|  | Euryarchaeota | Methanosarcinaceae | Methanimicrococcus | 1 | 0.45 | 0.005 | 0.001 | 0.003 | 1.79 |
| Eukarya (Fungi) | Fungi Unclassified |  | Fungi | 7 | 68.69 | - | 75.13 | 64.72 | 66.22 |
|  | Ascomycota |  | Ascomycota | 1 | 0.05 | - | 0.07 | 0.008 | 0.06 |
|  | Ascomycota | Eurotiomycetes | Byssochlamys | 1 | 0.84 | - | 0.004 | 2.50 | 0.004 |
|  | Ascomycota | Eurotiomycetes | Spiromastix | 1 | 1.77 | - | 0.03 | 0.29 | 4.97 |
|  | Basidiomycota | Malasseziomycetes | Malassezia | 1 | 0.01 | - | 0.013 | 0.01 | 0.004 |
